# Paradigms of intraoperative neuromonitoring in paediatric thyroid surgery

**DOI:** 10.3389/fendo.2024.1455217

**Published:** 2025-01-30

**Authors:** Yuchuan Li, Chunhai Zhang, Yi Zhang, Gianlorenzo Dionigi, Yishen Zhao, Hui Sun, Yingying Wang

**Affiliations:** ^1^ Department of Thyroid Surgery, China-Japan Union Hospital of Jilin University, Jilin University, Changchun, China; ^2^ Division of Surgery, Istituto Auxologico Italiano IRCCS (Istituto di Ricovero e Cura a Carattere Scientifico), Milan, Italy; ^3^ Department of Pathophysiology and Transplantation, University of Milan, Milan, Italy; ^4^ Jilin Provincial Key Laboratory of Surgical Translational Medicine, Jilin Provincial Precision Medicine Laboratory of Molecular Biology and Translational Medicine on Differentiated Thyroid Carcinoma, Changchun, China

**Keywords:** children, paediatrics, infant, adolescent, thyroid gland, recurrent laryngeal nerve, intraoperative neuromonitoring

## Abstract

The larynx of children and adolescents is still in the developmental phase and the anatomical structure is still very small and sensitive. The higher malignancy and faster progression of some paediatric thyroid cancers make surgery more difficult. Intraoperative neuromonitoring (IONM) is frequently used in thyroid surgery as an effective means of securing the recurrent laryngeal nerve (RLN). Little information is available on the clinical efficacy of IONM in paediatric surgery. In addition, classic IONM techniques such as reinforced tracheal tube models with integrated surface electrodes are not standardised for children and adolescents. The use of innovative devices such as laryngeal masks with surface electrodes and thyroid cartilage receiving electrodes could replace monitoring tubes as a new form of IONM. Tracheal intubation in children needs to be performed by a highly experienced anaesthetist. The continued maturation of AI technology could be attempted in the future in conjunction with IONM to further reduce RLN injuries in children and adolescents. This article describes the anatomical features of the paediatric larynx, which differ from those of adults, and the advantages and shortcomings of IONM techniques for thyroid surgery in this population. The use of IONM in paediatric surgery is a complex technique and should be performed by experienced thyroid surgeons with in-depth IONM training. The use of IONM should be standardised within the clinical parameters of children.

## Introduction

The guidelines for the treatment of thyroid nodules and differentiated thyroid cancer in children, published by the American Thyroid Association in 2015, highlight paediatric thyroid cancers with higher malignancy (1). In addition, children and adolescents have unique neck anatomy compared to adults, such as thinner recurrent laryngeal nerves (RLN), an ectopic thymus, and a more limited surgical space, which makes thyroid surgery more difficult to perform and increases the risk of postoperative complications. Intraoperative nerve monitoring (IONM) is a non-invasive tool that allows effective localisation and identification of nerve courses, detects nerve branches, anatomical deviations and indistinguishable tissues, avoids excessive anatomical excursions and provides real-time data to optimise intraoperative decision making. IONM was first introduced in the 1980s and has since been widely used in neonates, children and adults (2, 3).

Observational studies have shown that IONM is a more effective alternative to conventional, unsupervised surgery in paediatric thyroid surgery. Systematic reviews have provided evidence that IONM can be used effectively and safely in children. With increasing experience and acceptable efficacy, the use of IONM has been extended to other paediatric patients with a range of conditions including thyroid cancer, hyperthyroidism and lymph node removal (4–6).

Although IONM is now being used more frequently, there is limited information on the outcomes of IONM technology in paediatric patients. Exploring a proven method for IONM in thyroid surgery in children and adolescents with localised anatomical features of the neck is one of the current technical bottlenecks in this field and also represents the focus and difficulty of IONM in this population. The aim of this study was to investigate the technical characteristics and outcomes of IONM in paediatric patients undergoing thyroid surgery.

## Anatomy

### Characterisation of the RLN in the paediatric population

It is known that the left and right laryngeal nerves are asymmetrical, e.g. in males the left laryngeal nerve is on average 43 cm long and the right laryngeal nerve is on average 32 cm long. Jotz et al. found that the area and circumference of the right laryngeal nerve were positively correlated with body weight and BMI (P < 0.05), while the left laryngeal nerve showed no significant correlation (7). This feature is also present in the paediatric and adolescent population. By analysing data from Schneider’s neuromonitoring of 504 children, such as initial amplitude and latency, Diercks et al. concluded that intraoperative nerve monitoring (IONM) in adults is equally applicable to children (4, 8).

### Application of IONM in EBSLN

The external branch of the superior laryngeal nerve (EBSLN) mainly contains special visceral motor fibres that innervate the cricothyroid muscle. The location of EBSLN is relatively fixed at the sternothyroidlarynx triangle, which has the sternothyroid as its lateral border, the inferior constrictor of the pharynx and CTM as the medial borders, and the superior pole of the thyroid gland as the lower border. In addition to the elongated and variable EBSLN, factors such as high hyperthyroidism, oversized mass, short and thick neck, strong adhesion of the mass to the gland and secondary surgery are prone to its damage, resulting in a change in the patient’s tone and pitch (9–11). A survey of thyroid surgeons found that only 38.51% attempted to recognise it during surgery (10). IONM can detect EBSLN by stimulating the EBSLN to produce cricothyroid twitch or by receiving electromyographic signals. Between 0.3% and 58% of patients undergoing thyroid surgery are at high risk of EBSLN (12). EBSLN injury alters the innervation of the hypopharyngeal constrictor and cricothyroid motor nerves, unilateral injury may affect the tightness of the vocal folds, resulting in lower pitch, hoarseness, inability to raise pitch suddenly, etc., and bilateral injury to the timbre, the change in tone quality is more pronounced, there may be a monotonous timbre, etc. Severe impairment of quality of life in certain occupational groups (13). Luis et al. evaluated 240 cases of EBSLE with the naked eye or IONM in 148 patients and found that IONM identification was better than visual identification alone (13). Using neuromonitoring, Meyer et al. found that IONM was equally suitable for the detection of EBSLN in paediatric thyroid surgery (14). The rate of transient EBSLE injury with IONM was 58% and the rate of permanent injury was 3.8% (15). The detection rate of EBSLN was 34% without IONM and 84% with IONM (16). However, there are only a few reports on children (8, 14).

### Characteristics of thyroid cancer in children

Thyroid surgery in children and adolescents is unique not only because of its anatomy, but also because of its degree of malignancy and prognosis compared to other diseases. In a Dutch national study, a population of children with differentiated thyroid cancer had an overall survival rate of 99.4% after a median follow-up of 13.5 years (17). Patients who live longer endure the psychological effects of daily calcium supplementation and changes in daily communication (18). Children and adolescents have higher rates of malignancy, bilateral morbidity and cervical lymph node metastases in thyroid nodules compared to adults. The rate of extracapsular spread in children compared to adults is 50% versus 30%, regional lymph node involvement is 80% versus 50% and distant metastasis is 30% versus 5% (19). In a study by Banic et al. of 212 children (mean age 14.1 years) with papillary thyroid cancer (PTC), bilateral multifocal disease was found in 73 (34%) patients (19). Analysis of 52 pre-school (<7 years) and school-age (8-13 years) PTC patients by Chen et al. showed that the pre-school group had more local invasion (P=0.01), higher tumour stage and postoperative complications (P<0.05) (20). In a study of 740 children and adolescents (mean follow-up time 115.8 months), a recurrence rate of 27.6% was found (21). Childhood thyroid cancer is an unusual solid tumour, and like adults its different subtypes have different invasive characteristics. Studies have shown that PTC in children does not have the typical papillary morphology of adult PTC and is extensively infiltrated throughout the gland with follicular and solid structures. Follicular thyroid cancer (FTC) in children and adolescents has a much lower incidence and is often a single lesion; most patients with FTC have a good prognosis, but there are still some FTC that are prone to haematogenous metastases in the early stages (1). Medullary Thyroid Carcinoma (MTC) in children and adolescents is hereditary, and studies have found that the rate of MTC lymph node metastasis in children and adolescents is lower than in adults (P < 0.05), but twice as multifocal (22). Due to the different characteristics of different subtypes, some of which are highly invasive and multifocal, complications during surgery can be reduced with the help of IONM. Children and adolescents are in the developmental phase, have finer nerves compared to adults, the surgical area is smaller, the recurrent laryngeal nerve is covered by ectopic cervical thymus tissue, the operation is difficult and lengthy, the blood vessels are thin and weak, and at the same time the anatomical skill of the surgeon is extremely high, a national study in the Netherlands found that lifelong complications occurred in 32.4% of paediatric patients (17, 23). The risk of bilateral RLN injury in children was reported to be 1.5% (4). The use of IONM can better identify and assist the surgeon in recognising the nerves in paediatric and adolescent surgery, shorten operative time and reduce intraoperative and postoperative complications. It is also helpful for low-volume surgeons and young surgeons to quickly master thyroid surgery (24).

## Vocal cord paralysis in paediatric population

RLN injury is the most common complication of thyroid surgery. The RLN rate after total thyroidectomy in adults is about 3.5% for transient lesions (25). In children, the rates of transient lesions and definitive lesions after thyroidectomy are 1.55-8.0% and 2.5% respectively (24, 26). The rate of RLN lesions continues to decrease with increasing age of the child (27). In children, the surgical structures are smaller, particularly the nerve diameter, and the rate of cancer is higher than in adults. As a result, surgery is more risky in this age group.

## Pathology

### Clinical features and surgical approaches to thyroid cancer in childhood and adolescence

Thyroid cancer in childhood accounts for 4-5% of childhood cancers, with an incidence rate of 0.5 cases/100,000; the incidence rate in adolescents is ten times higher than in younger children (1), compared with an incidence rate of 2-10/100,000 in adults (6). The proportion of malignant thyroid nodules in children is relatively high, with a malignancy rate of 22% to 26% (1). The RLN has many different branches, which are formed by anatomical variations and have different characteristics depending on gender and ethnicity. It is very difficult to recognise and protect all branches of the RLN with the naked eye in conventional surgery. Fontenot et al. studied 491 patients who underwent thyroid surgery, 405 women and 86 men; 218 African Americans, 251 Caucasians and some other ethnic groups, and found that the distance from the branching point of the RLN to the entry point into the larynx was greater in female patients than in male patients (11.04 mm vs. 8.56 mm), and that the rate of branching of the RLN was higher in African-Americans than in Caucasians (42.1% vs. 33.2%), while the distance from the branch to the laryngeal entrance was not significantly different. It is worth investigating whether children and adolescents have unique characteristics such as age patterns in addition to gender and ethnicity (28).

Children and adolescents should require a complete thyroid ultrasound, thyroid fine needle aspiration and BRAF genetic testing prior to thyroid surgery. Preference can be given to scarless neck surgery such as lumpectomy or da Vinci robot without compromising prognosis (29).

### Laryngeal development and the choice of tracheal intubation in children and adolescents

Understanding laryngeal development in children and adolescents facilitates the development of appropriate catheter-based monitoring electrodes for patients of different ages. In infants, the larynx is funnel-shaped (30), with the level of the subglottic cricoid cartilage being the narrowest part. The adult larynx is cylindrical, with the narrowest part at the opening of the vocal folds (31). In newborns, the vocal folds correspond to the height of cervical vertebrae 3-4 (in adults to the height of cervical vertebrae 5-6). At the age of 6 years, the vocal folds descend to the level of cervical vertebrae 5, and at the age of 13 years they reach the human position. In the course of adult development, the larynx gradually takes on a cylindrical shape (32, 33). In prepubertal children, there is no significant difference in vocal fold length between males and females, whereas with the onset of puberty, the extent of vocal fold growth in males more than doubles that of females, with vocal fold length increasing by 64 in males but only 34% in females, and the length of vocal folds of both sexes essentially reaching adult size after puberty (34). In females, the internal diameters of the subglottis and cricoid cartilage increase linearly with age until adulthood, whereas in males, the internal diameters of the subglottis and cricoid cartilage increase rapidly between the ages of 12-15 years. The choice of type of tracheal intubation in children and adolescents is mainly based on age-based formulae such as the Cole formula and Motoyama formula, in addition to related formulae based on height and weight, but the actual results are suboptimal due to differences in airway development in children and adolescents, and ultimately multiple repeat laryngoscopies are still required (35).

In children and adolescents, the selection of an appropriate endotracheal tube (ETT) is very important. Too small an inner diameter of the tube leads to inadequate ventilation, poor reliability of end-expiratory gas monitoring, leakage of anaesthetic gases and increases the risk of aspiration, while too large an inner diameter of the tube not only increases the risk of upper airway injury, such as ischaemic oedema of the local mucosa, but also increases the difficulty of intubation (35). It has therefore been suggested that the appropriate diameter of the tracheal tube should be determined using ultrasound images. The measurement of the subglottic diameter at the level of the cricoid cartilage by ultrasound allows a targeted selection of the ETT (36). Dalal et al. used a bronchoscopic technique to measure laryngeal dimensions in 135 children under general anaesthesia and found that the cricoid cartilage was oval rather than round (33). Uncuffed round ETTs lead to leakage in the anterior and posterior interspaces of the airspace and excessive pressure on both sides, whereas cuffed ETTs reduce the risk of air leakage during positive pressure ventilation and the risk of air contamination of anaesthetic gases and are therefore increasingly used in paediatric surgery (37). Litman et al. (38) found from laryngeal measurements in 99 children aged 2 monthsto 13 years that in sedated, non-paralysed children, the narrowest portions of the larynx were the glottic opening (at the level of the vocal folds) and at the level of the subglottis, and that the larynx was frequently injured in children who were intubated and mechanically ventilated for long periods, with the worst damage often occurring at the level of the vocal folds.

Tracheal intubation in children is challenging, with studies finding that only two-thirds of patients can be successfully intubated on the first attempt, with the most common challenge being the obscuring of the laryngeal view by body night (39). The table below lists some of the common problems associated with tracheal intubation in children and the paediatrician’s experience in resolving them (39) (**Table 1**).

**Table 1:** Difficulties of pediatric intubations and the experience of Pediatric endocrine surgeons.

Challenge	Experience with solutions	References
Coughing or crying	Parents present or preoperative sedative medication	(40)
Body fluids obscure the view of the larynx Multiple failed intubations Endobronchial tube Bradycardia/hypotension	Airway suction, repositioning the patient Replacement of equipment, replacement of operators Use of an electronic laryngoscope or fibreoptic bronchoscope Use of muscle blockers in mild or moderate anaesthesia	(39) (39) (41) (42)

The anatomical characteristics of the larynx in children and adolescents therefore make it necessary to select different tracheal tube models for intubation depending on gender and age group in order to avoid intubation-related vocal cord injuries. In neonates, younger children and children with airway obstruction, if a tracheal tube model is no longer suitable, the operator should not rely on ETT electrodes and try other modalities. Cervical skin adhesion electrodes and thyroid cartilage electrodes are used as novel methods of neuromonitoring. In one study, 12 piglets were introduced to neuromonitoring, and electromyographic signals induced by electrodes on the surface of the ETT and the skin of the anterior cervical region were recorded, and electromyographic signals of the recurrent laryngeal and vagus nerve stimulated by a current of 1 mA were successfully recorded in all cases (43). In a porcine model of the recurrent laryngeal nerve, we compared adhesive thyroid cartilage electrodes (ATECs) with conventional ETT electrodes and found that ATCEs had consistent monitoring function with ET electrodes and needle electrodes. Compared with needle electrodes, atce is a noninvasive procedure, and in contrast to ETT electrodes, atce did not affect EMG signals due to changes in endotracheal tube position during the procedure (44). In addition, there are still doctors through the visual laryngoscope in the operation through the space in the laryngeal mask to observe the changes in the movement of the vocal cords, which is also an indirect reflection of the function of the RLN in the operation of a kind of exploratory ideas.

## IONM technology

### IONM protocols

In addition to the tracheal tube recording electrodes for neuromonitoring, there are also adhesive electrode sheets, needle electrodes, etc. The various recording electrodes can be used for patients of different age groups and different tracheal calibres. In patients undergoing secondary surgery, the surgical site is often altered by cryosurgical scars, the anatomy is disturbed, and there is a greater likelihood of nerve-tissue adhesions that make visualisation of the recurrent laryngeal nerve difficult (45). In these cases, IONM can better support the surgeon in neuromonitoring the surgical procedure. IONM is also excellent for identifying the non-recurrent laryngeal nerve (NRLN), which is most common on the right side with an incidence of 0.3%-0.8% (46–48). The NRLN can be easily injured if it is not recognised in time during thyroid surgery or if the dissection process or the surgeon are inexperienced. Preoperative enhanced CT of the neck can be used to determine the likelihood of the presence of an NRLN preoperatively by identifying the aberrant right subclavian artery (49). However, due to the radiation risks of CT, it is unsuitable as a routine preoperative examination for children and adolescents. A study (50) by Brauckhoff et al. in 18 adults with NRLN found that in IONM, a latency of less than 3.5 ms after vagal stimulation indicates the presence of NRLN prior to dissection, and that this data is reduced to 2-3 ms in paediatric and adolescent patients with lower BMI.

### Characteristics of intraoperative neuromonitoring techniques in children and adolescents undergoing thyroid surgery

In thyroid surgery, the likelihood of recurrent laryngeal nerve injury is greater in children than in adults (4). Standardised use of IONM can significantly reduce the risk of recurrent laryngeal nerve injury. Strict adherence to the IONM guidelines and the consensus of relevant experts can effectively protect the function of the RLN (4, 11). Vocal cord paralysis is a common complication of thyroid surgery. Bergenfelz et al. (51) found that the use of IONM reduces the risk of permanent vocal cord paralysis. Laryngeal recurrent paralyses occur in children and adolescents with a probability of 1% to 18.8% (52). In a controlled study of IONM in children and adolescents, Ritter et al. (24) found that the risk of RLN palsy is higher at a younger age and therefore advocated that the IONM technique should be considered in children under 10 years of age, particularly for procedures requiring simultaneous cervical lymph node dissection. Brauckhoff et al. (26) performed thyroidectomy in 97 children (mean age 11.7 years), 53 of whom underwent intraoperative neuromonitoring and 44 in the control group did not receive intraoperative neuromonitoring; postoperatively, they found that 1.89% of the neuromonitoring group had transient vocal cord paralysis; 4.47% of the control group had transient vocal cord paralysis and 2.27% had permanent vocal cord paralysis. The use of IONM to minimise RLN injury is well documented, and IONM not only identifies the site of injury in a timely manner, but also determines the extent of injury based on electromyography. This is of great importance for children and adolescents, e.g. in the case of bilateral total thyroidectomy, when the question is whether or not to perform staged surgery after one side of the nerve has been damaged.

The choice of type and dose of anaesthetics and muscarinic agents is also a challenge in intraoperative IONM in children and adolescents as they affect neuromuscular junction and synaptic function. Propofol is a commonly used anaesthetic in IONM, and targeted infusion can be achieved in children and adolescents by using the Paedsufor and Kataria models. Muscle relaxants act at the neuromuscular junction and block impulse transmission between neuromuscular muscles. Moderate amounts of muscle relaxants can be used to fulfil the requirements of tracheal intubation without compromising the strength of the neuromonitoring signal (2).

### Features of the laryngeal mask in combination with a fibreoptic laryngoscope for neuromonitoring

Laryngeal mask ventilation was first proposed by the British scientist Brain in 1963. When anaesthetising patients via the laryngeal mask, an electronic laryngoscope can be connected to the laryngeal mask to electrically stimulate the target nerves, and the RLN can be identified and functionally assessed by observing the movement of the vocal cords through the electronic laryngoscope, which is effective in monitoring intraoperative neurological function in children and adolescents (53). The laryngeal mask to assess RLN function reduces postoperative dysphagia and vocal cord dyskinesia due to balloon dilatation of the catheter compared to conventional monitoring catheters. The incidence of postoperative sore throat was 47% for procedures with conventional endotracheal intubation and was even higher for monitoring catheters with bare metal electrodes at the end, while the sore throat caused by the laryngeal mask was only 7% (53). Hillermann et al. (54) Thirty patients undergoing thyroid surgery underwent laryngeal mask, vocal cord movement monitoring and observation of vocal cord movement and postoperative recovery of the patients, and only in one case was transient damage to the RLN observed. Neuromonitoring with a laryngeal mask in combination with fibreoptic laryngoscopy is also a viable option in younger children with even smaller tracheal diameters where monitoring catheters are difficult to insert. However, the sample size of the above report is limited and needs to be validated by a large number of clinical studies. In addition, the existing laryngeal mask combined with vocal cord movement testing requires continuous observation of vocal cord movement, and if vocal cord movement is impaired, the RLN is already severely damaged.

### Thyroid cartilage needle electrodes in intraoperative neuromonitoring in children and adolescents

The needle electrode method is an altrernative recoding-side system that provides nerve monitoring over the thyroarytenoid muscle (55). When monitoring 205 high-risk RLNs with ETT electrodes and TC electrodes, Chiang et al. found that TC electrodes not only had the same stability as ETT electrodes, but the measured EMG signals were also more stable (56). In an animal experiment in which piglets were selected for both tracheal tube neuromonitoring and needle electrode monitoring, nine zones were divided on each side of the thyroid cartilage and neuromonitoring was performed with needle electrodes in different zones to investigate the feasibility of needle electrodes for obtaining laryngeal extension signals (57). This also provides a basis and reference for the location of needle electrodes for monitoring in children and adolescents. Zhao At-Shen et al. applied thyroid cartilage needle electrodes and tracheal tube surface electrodes simultaneously in 136 patients undergoing thyroid surgery and found that the amplitude of EMG signals from thyroid cartilage needle electrodes was more significant (P < 0.05), with less disturbed EMG signals, and there was no influence of EMG signals due to tracheal tube deflection. TC electrodes get rid of the dependence on the tracheal tube and can effectively avoid the instability of electromyographic signals caused by the disadvantages of ETT electrodes, such as the lack of tracheal tube rotation, deep or shallow intubation; it is not necessary to completely and accurately match the catheter electrodes to the vocal cords; it can be used in patients with tracheal deformities or large tracheal goitre compression; and the use in non-anticipatory surgery is the advantage of its use. Of course, TC electrodes have certain limitations, such as the placement of TC electrodes must be free of thyroid cartilage, which is less suitable for small incisions, luminal and robotic surgery; TC electrode placement is an invasive procedure that can cause complications such as haematoma and infection. In children and adolescents, TC electrodes may become a new alternative to ETT electrodes, but the thyroid cartilage is thinner in children and adolescents than in adults, and the appropriate electrode insertion depth, insertion position and probe size need to be further researched.

### Perspectives of artificial intelligence in thyroid surgery in children and adolescents

Artificial intelligence (AI) has come to the fore at the beginning of the 21st century (58). The field of medicine has developed significantly in recent years. AI has been shown to be useful in all aspects of the diagnosis and treatment of thyroid disease. Peng et al. (59) developed an AI model for detecting the type of nodules in thyroid ultrasound images, and using this model, the combined AUC of specialists improved from 0.837 to 0.875 (p < 0.001). Current AI models can also determine the subtype of thyroid cancer in postoperative paraffin wax (60). For intraoperative detection of the laryngeal return nerve, the AI model trained by Gong et al. performed well in detecting RLNs in open surgical images (61). During surgery, EMG signals from the IONM change in real time, and the observer and surgeon may not communicate in time when problematic signals occur, which may lead to nerve damage. Inexperienced observers who do not react immediately to problematic signals can also lead to nerve damage. The AI model developed by Zha et al. enables the automatic identification of electromyographic signals from intraoperative IONM in thyroid surgery with an accuracy of 89.54% (62).

All of the above results can be utilised in the diagnostic and therapeutic process of thyroid disease in children and adolescents to reduce leakage, misdiagnosis and perioperative complications. The combination of IONM with proven AI during surgery will undoubtedly improve the success rate of RLN protection. In children and adolescents, AI during lumpectomy or robotic thyroid surgery could be considered in the future to train a neural alignment prediction model after collecting enough neural alignment data sets, which would further improve RLN protection.

### Auxiliary control of the posterior cricoarytenoid muscle

The posterior cricoarytenoid (PCA) is the only abductor muscle that controls the vocal folds. Routine neuromonitoring focuses on the internal laryngeal retractor muscles that control the vocal folds. Some studies have combined a concave bipolar electrode with a gastric tube or placed the bipolar electrode directly at the PCA using a laryngoscope, where the weight of the laryngeal structures holds the electrode in place (63). Simultaneous monitoring of both the internal retractor and the posterior cricoarytenoid muscles controlling the vocal folds allows better observation of intraoperative RLN function and prediction of postoperative airway patency. Liddy et al. (64) monitored patients simultaneously with PCA electrodes and tracheal tube surface electrodes and found that the mean EMG amplitude of the PCA was 45.4% and 53.1% of the ipsilateral acoustic amplitude during vagal and RLN stimulation, respectively, providing some indication of the normal EMG parameters of the PCA. PCA monitoring may also be useful in analysing the RLN branching components. It is routinely assumed that the RLN is located in the anterior branch when the RLN branches, but Barczynski et al’s analysis of 2,500 RLNs found that the motor branch of the RLN was located in the posterior branch in eight cases, and vocal cord abduction can be assessed by palpation of the posterior cricoarytenoid muscle (65).

PCA monitoring has not been studied in children and adolescents, probably because of the additional equipment required and the higher number of surgeries, but PCA monitoring can be used in conjunction with other neuromonitoring devices to predict vocal fold activity as well as to troubleshoot catheter electrodes when the signal is lost due to catheter deflection, and it can be used to analyse the branching components of the RLN, which is promising for use in surgery in children and adolescents.

### Inadequacies of current neurological monitoring techniques for children and adolescents

Various models of recording electrodes are available today, the most commonly used being mainly endotracheal tubes with integrated surface electrodes, tracheal tubes with adhesive electrodes and posterior cricoid cartilage adhesive electrodes.

**Figure 1:**
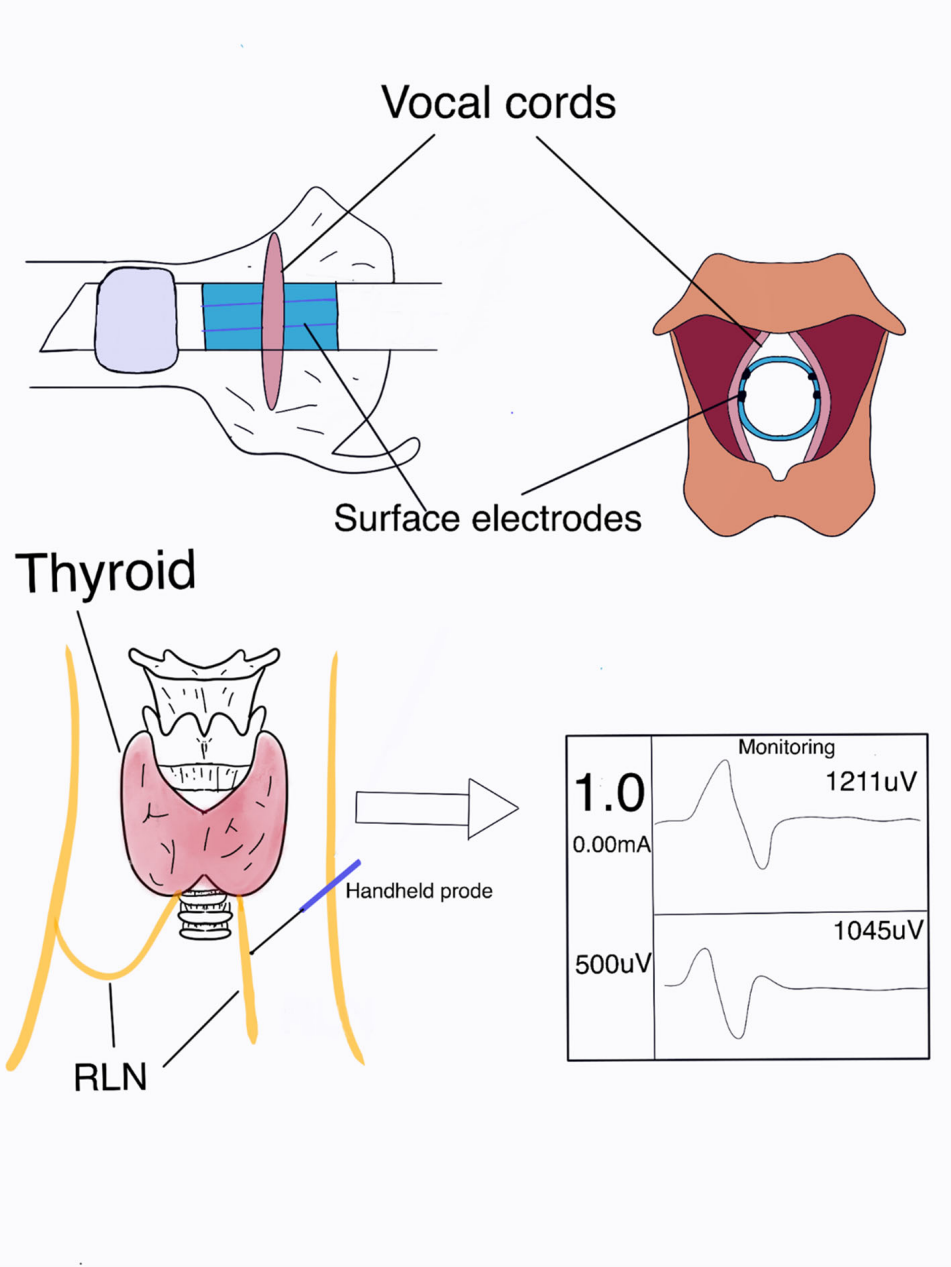
Neuromonitoring demonstration.

Endotracheal catheters with integrated surface electrodes are the most commonly used and are non-invasive recording electrodes. In Europe and the United States, the optimal tracheal catheter size (the catheter size is the inner diameter of the catheter) is 7.0 for adult females and 7.5 for males. In the Asian population, the most appropriate model is 7.0 for adult males and 6.5 for females. The following table refers to Europe and the United States (**Table 2**) (4). Below is a demonstration of tracheal catheterized neuromonitoring (**Figure 1**) and a flow chart of routine neuromonitoring (**Table 3**).

**Table 2:** Adaptation of tracheal catheter models to age in children in Europe and the United States.

Tracheal tube model	Age
5.0	5-8
6.0	9-12
7.0	13-18

In China, there are no detailed standards for neuromonitoring catheters for children and adolescents of different ages. There are no standards for the type of neuromonitoring catheters for children and adolescents at different stages of development. Larger catheters may cause more complications, while smaller catheters may lead to unstable fixation of the catheter, making it susceptible to deflection and resulting in inaccurate neuromonitoring readings.

**Table 3:** Standardised procedures for neuromonitoring: routine examination of the vocal cords and equipment prior to monitoring. Intraoperatively, the four steps of monitoring were followed and the EMG signals were recorded and interpreted.

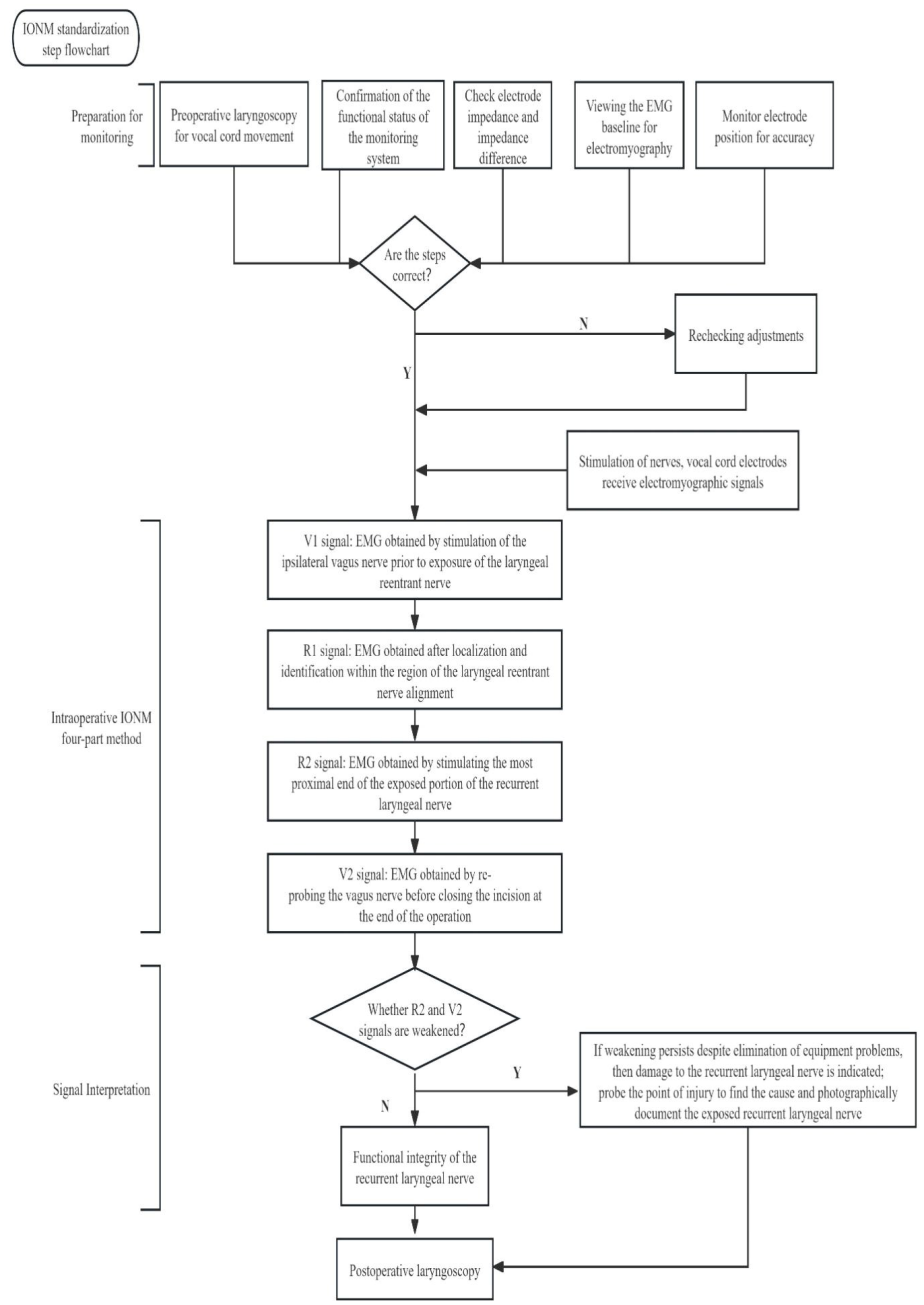

Needle electrodes are needle electrodes that are inserted under the thyroid cartilage into the vocal cord muscle, which receives a stable EMG signal and records more accurate data. A study (66) of RLN monitoring by bipolar electroacupuncture of the cricothyroid ligament in nine thyroid operations in children and adolescents found no postoperative complications and the specificity of the monitoring was 100%. Boise et al. (67) successfully monitored 15 RLNs by inserting a double needle electrode through the cricothyroid membrane and no vocal cord abnormalities occurred postoperatively, with the exception of one nerve that was transected due to malignant invasion. Needle electrodes are an invasive procedure that can result in local tissue damage, haematoma, broken electrodes left in the body or displacement of the electrodes. However, the signal stability of the needle electrode is stronger than that of the tracheal tube electrode, and the signal does not become unstable due to rotation of the tube, poor contact with the vocal cords or too deep or too shallow intubation, making it a reliable tool for the operator.

The adhesive electrodes can be placed 1-2 cm above the airbag for tracheal intubation positioning, the bilateral surface electrodes are arranged in pairs and symmetrically distributed on the surface of the tube wall, and the neuromonitoring catheter with the corresponding diameter can be selected according to the conditions of different patients, which is beneficial to the operation of children and adolescents. However, good contact between the surface electrodes of the monitoring catheter and the vocal cords is required, and rotation and inaccurate placement of the monitoring catheter may affect the accuracy of EMG signal recording. The use of adhesive electrodes had no effect on the incidence of postoperative pharyngeal complications (68).

With the development of IONM technology, continuous neuromonitoring technology has also been applied, in which the jugular arteriovenous sheath is cut prior to thyroid manipulation, the continuous monitoring electrode (APS) is attached to the vagus nerve boot, and current is automatically released to stimulate the vagus nerve for real-time monitoring of laryngeal recurrent nerve function. Schneider et al. (69) performed thyroid surgery in 258 children and adolescents with continuous and intermittent nerve testing, respectively, and found that nerve damage and vocal cord paralysis were only observed with intermittent nerve testing, suggesting that the continuous nerve testing technique may be more beneficial in preventing early and permanent vocal cord paralysis. This is supported by Schneider et al. who analysed the incidence of postoperative vocal cord paralysis in 3139 consecutive IONM and 2890 interrupted IONM procedures (70). APS can be used in younger children, with the youngest patient being 5 months and 9 days old. This technique has some disadvantages, such as the need to open the carotid sheath, and the continuous stimulation of the vagus nerve may lead to unwanted stimulation of the vagus nerve (71). For the APS electrodes, the older the child, the better the results, probably due to the better fit of the device in older children. The median baseline amplitude increased with the age of the child (69). Studies have shown that continuous neuromonitoring may be more effective in reducing risks such as nerve palsies compared to routine intermittent neuromonitoring. The study by Schneider et al. on children and adolescents undergoing thyroid surgery found that all patients who developed transient vocal cord paralysis after surgery were in the intermittent neuromonitoring group (69).

Continuous IONM allows real-time assessment of nerve function, effectively solving the untimely monitoring of nerves by ill-equipped hands leading to nerve injury, and also reducing nerve injury due to excessive pulling of the nerve by the surgeon during the procedure (**Table 4**).

**Table 4:** Thyroid surgery cases for children and adolescents.

Article Source	Total sample size	Sample size for each group	Age (years)	Sex (M: F)	Partial thyroidectomy or other surgery	Total thyroidectomy	No monitoring	With neuromonitoring	Catheter inner diameter (mm)	Laryngeal recurrent nerve injury/strip (temporary)	Unnecessary laryngeal nerve injury/strip (permanent)
Martucci et al (5)	25	12	3.9-18	6:06	3	9	Y	N	6.0-8.0	1	0
		13	1.5-13.9	3:10	4	9	N	Yes (tracheal tube surface electrode)	6.0-8.0	1	0
Legre et al (66)	47	10	3-15.5	—	6	4	Y	N	4.7-6.3	1	1
		28	10.5-18	—	18	10	N	Yes (tracheal tube surface electrode)	5.8-6.4	3	0
		9	0.6-15	—	3	6	N	Yes (needle electrode)	4.91-5.09	2	0
Daniel et al (72)	307	169	4-21	69:238	96	211	N	Y	—	4	0
		138					Y	N	—	1	0
Bois et al (67)	9	—	Average 4.7	6:03	3	6	N	Y (double needle electrode)	3.5-6.0	0	0
Ritter et al (24)	113	36	9-19	9:27	21	15	N	Y	—	1	0
		77	2-19	20:57	43	34	Y	N	—	5	2
Propst et al (73)	26	—	4.5-17.4	9:16	—	—	N	Y (adhesive electrode, integrated electrode)	4.0-7.5	—	—
Schneider et al (69)	258	210	0-18	69:141	62	148	N	Y (intermittent IONM)	—	6	1
														48	0-18	19:29	13	35	N	Y(APS IONM)	—	0	0

## Conclusion

In contrast to interventions in adults, the range of indications more frequently includes nodules suspected of being malignant, autoimmune thyroidectomies, prophylactic thyroidectomies for genetic mutations and thyroglossal duct cysts. When determining the indication, particular attention must be paid to the anatomy, physiology and the increased risk of complications (especially hypoparathyroidism). Thyroid surgery in children and adolescents must be performed by surgeons who are particularly experienced in thyroid surgery and who perform a high number of operations. The anaesthetist performing the tracheal intubation should also be experienced. The use of innovative devices such as laryngeal masks with surface electrodes and thyroid cartilage receiving electrodes is a new form of IONM. The problems in surgical procedures are mainly related to the difficulties in identifying the RLN, which is smaller than in adults and can be covered by the ectopic cervical thymus tissue, as the collateral RLN fibres innervate the thymus. The child’s larynx and trachea are smaller than in adults. The overall laryngeal structure is softer in children and, although less susceptible to blunt trauma, is more prone to collapse due to the negative inspiratory pressure generated during breathing. The use of a magnifying device (magnifying binocular or surgical microscope) and IONM ensures better identification of reliable neck structures.
